# Biological Markers to Predict Outcome in Mechanically Ventilated Patients with Severe COVID-19 Living at High Altitude

**DOI:** 10.3390/jcm12020644

**Published:** 2023-01-13

**Authors:** Jorge Luis Vélez-Páez, Paolo Pelosi, Denise Battaglini, Ivan Best

**Affiliations:** 1 Facultad de Ciencias Médicas, Universidad Central de Ecuador, Quito 170129, Ecuador; 2 Laboratorio de Inmunología, Facultad de Ciencias y Filosofía, Departamento de Ciencias Celulares y Moleculares, Universidad Peruana Cayetano Heredia, Lima 15074, Peru; 3 Unidad de Terapia Intensiva, Hospital Pablo Arturo Suárez, Centro de Investigación Clínica, Quito 170129, Ecuador; 4 Department of Surgical Sciences and Integrated Diagnostics, University of Genoa, 16132 Genoa, Italy; 5 Anesthesiology and Critical Care, San Martino Policlinico Hospital, 16132 Genoa, Italy; 6 Carrera de Medicina Humana, Facultad de Ciencias de la Salud, Universidad San Ignacio de Loyola, Lima 15024, Peru

**Keywords:** SARS-CoV-2, coronavirus infection, mortality, biomarkers, COVID-19

## Abstract

Background: There is not much evidence on the prognostic utility of different biological markers in patients with severe COVID-19 living at high altitude. The objective of this study was to determine the predictive value of inflammatory and hematological markers for the risk of mortality at 28 days in patients with severe COVID-19 under invasive mechanical ventilation, living at high altitude and in a low-resource setting. Methods: We performed a retrospective observational study including patients with severe COVID-19, under mechanical ventilation and admitted to the intensive care unit (ICU) located at 2850 m above sea level, between 1 April 2020 and 1 August 2021. Inflammatory (interleukin-6 (IL-6), ferritin, D-dimer, lactate dehydrogenase (LDH)) and hematologic (mean platelet volume (MPV), neutrophil/lymphocyte ratio (NLR), MPV/platelet ratio) markers were evaluated at 24 h and in subsequent controls, and when available at 48 h and 72 h after admission to the ICU. The primary outcome was the association of inflammatory and hematological markers with the risk of mortality at 28 days. Results: We analyzed 223 patients (median age (1st quartile [Q1]–3rd quartile [Q3]) 51 (26–75) years and 70.4% male). Patients with severe COVID-19 and with IL-6 values at 24 h ≥ 11, NLR values at 24 h ≥ 22, and NLR values at 72 h ≥ 14 were 8.3, 3.8, and 3.8 times more likely to die at 28 days, respectively. The SOFA and APACHE-II scores were not able to independently predict mortality. Conclusions: In mechanically ventilated patients with severe COVID-19 and living at high altitude, low-cost and immediately available blood markers such as IL-6 and NLR may predict the severity of the disease in low-resource settings.

## 1. Introduction

Biomarkers can be helpful for prognostic enrichment and for testing the efficacy of therapies according to biological sub-phenotypes in acute respiratory distress syndrome (ARDS) [1,2,3]. In patients with severe coronavirus disease 2019 (COVID-19), the search for biomarkers associated with clinical progression and prognosis could be considered as a possible option to clarify the evaluation, severity, and therapeutic management processes. Ferritin [4,5] and interleukin-6 (IL-6) [6,7,8,9] have shown their clinical utility; however, their high cost and the use of specialized equipment for the analysis may limit their use and clinical applicability in low-resource settings. Both inflammatory and hematological markers have been evaluated in series of patients with COVID-19 and various conditions of severity [4,7,10,11,12,13,14,15,16], although there is less evidence in critically ill patients undergoing invasive mechanical ventilation, as well as those patients that live at high altitudes. Some studies suggest lower mortality and severity rates of SARS-CoV-2 infection at higher geographical altitudes, probably due to acclimatization to hypobaric hypoxia and other determinants that have not yet been clarified [17,18]. Previous studies in critically ill patients with COVID-19 living at high altitude showed that interleukin-6 (IL-6) together with the neutrophil/lymphocyte ratio (NLR) and lactate dehydrogenase (LDH) were independent predictors of mortality [8]. The objective of this study was to determine the predictive value of inflammatory and hematologic markers on the risk of mortality at 28 days in patients with severe COVID-19 under mechanical ventilation and admitted to the intensive care unit (ICU), at high altitude and in a low-resource setting.

## 2. Materials and Methods

### 2.1. Study Design and Criteria of Inclusion

This was a retrospective observational study including patients with severe COVID-19 admitted to the ICU from 1 April 2020, to 1 August 2021. The study was approved by the ethics committee of the Universidad Peruana Cayetano Heredia, Lima, Peru (Code N° 301-30-21), and performed in the Pablo Arturo Suárez General Provincial Hospital located in Quito, Ecuador, at 2850 m above sea level (m.a.s.l.), which is an exclusive care center for symptomatic respiratory patients with COVID-19 who require hospitalization. A confirmed case of COVID-19 was defined in the presence of a nasal swab with a positive real-time reverse transcription polymerase chain reaction (RT-PCR) test. The inclusion criteria were the following: (1) age older than 18 years; (2) admission to the ICU requiring invasive mechanical ventilation. The study participants were retrospectively classified as survivors and non-survivors at the time of discharge from the ICU. Informed consent was waived due to the retrospective nature of this study in accordance with local regulations.

### 2.2. Data Collection

Information was collected from the electronic clinical records on clinical–epidemiological variables, including age, gender, comorbidities (diabetes mellitus (DM), arterial hypertension, obesity), clinical scales of organ failure and severity such as the sequential organ failure assessment (SOFA) and acute physiology and chronic health evaluation (APACHE) II, as well as inflammatory (D-dimer, ferritin, LDH, and IL-6) and hematological markers (mean platelet volume (MPV), NLR, MPV/platelet ratio). These variables were obtained at 24 h from admission to the ICU and in subsequent controls, and when available at 48 and 72 h. Routine blood counts and MPV values were measured using an automated hematology analyzer (Advia 2120i, Tarrytown, NY, USA), while ferritin and IL-6 were evaluated via chemiluminescence testing (Inmulite 2000 XPi, Malvern, PA, USA). LDH was measured via photometry (Advia 1800, Malvern, PA, USA) and D-dimer via enzyme-linked immunosorbent assay (ELISA).

### 2.3. Statistical Analysis

No formal sample size calculation was performed due to the exploratory, descriptive, and retrospective nature of the study. The variables are reported as medians (1st and 3rd quartiles) or absolute or relative frequencies (percentages) as appropriate. The Shapiro–Wilk test was used to assess the normal distribution of the data. For the quantitative variables, the Student’s *t*-test for independent samples or the Mann–Whitney test for a comparison between survivor and non-survivor groups was used as appropriate. The estimation of any association between the laboratory variables and survivors versus non-survivors was assessed with a preliminary univariate analysis (Chi-square test with Yates correction or Fisher’s exact test), followed by a multivariate logistic regression model adjusted for all baseline variables. Significant variables to the binary logistic regression model were entered in the multivariate model with the odds ratio (OR) and the 95% confidence interval (CI) as the main outputs. Statistical significance was established at *p* < 0.05. All statistical analyses were performed using R software version 4.1.2.

## 3. Results

### 3.1. Demographic and Clinical Characteristics of Patients with Severe COVID-19

**Figure 1:**
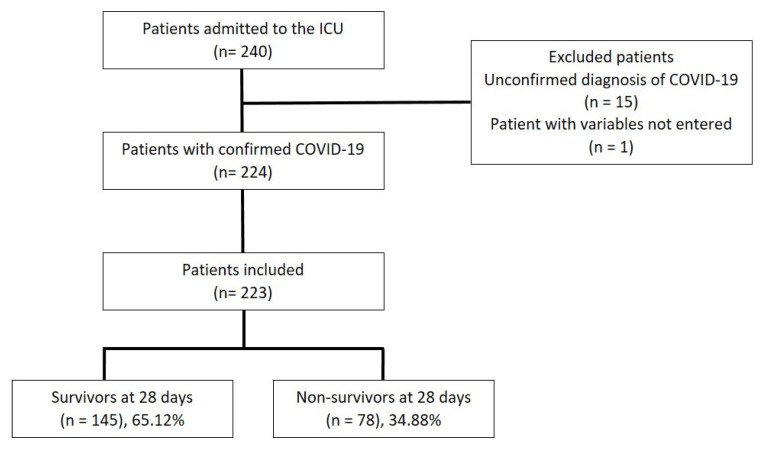
Flowchart of study inclusion and exclusion criteria. ICU: intensive care unit.

Overall, 240 patients were assessed for eligibility. Of these, 17 patients did not meet the inclusion criteria due to having an unconfirmed diagnosis and variables not being entered. Therefore, 223 patients were included in this study. At the time of ICU discharge, 145 (65.1%) patients survived, and 78 patients had died (34.9%) (Figure 1).

The median (1st quartile (Q1)–3rd quartile (Q3)) age of all patients was 51 (26–75) years. The non-survivors were significantly older (median age (Q1-Q3) years, 56 (31–79) years) than the survivors (48 (25–72) years) (*p* = 0.000). The most frequent comorbidity was obesity, followed by hypertension and diabetes mellitus, without differences between survivors and non-survivors. At ICU admission, a significantly higher APACHE II score was found in non-survivors compared to survivors (*p* = 0.010). SOFA, at 24, 48, and 72 h after ICU admission, was significantly higher in non-survivors compared to survivors (*p* < 0.001). In both survivors and non-survivors, the trend of SOFA scores showed higher values at 24 h, with decreasing values at 48 and 72 h. On average, corticosteroids were used in 90.1% of the patients, and 77.5% required low molecular weight heparin (LMWH) as an anticoagulant. The median (Q1–Q3) hospital stay was 10 (6–15) days, with no significant differences between survivors (10 (6–14)) and non-survivors (12.5 (6.8–12.3)) (*p* = 0.129). Table 1 shows the demographic and clinical characteristics of the overall population, survivors, and non-survivors in the ICU.

### 3.2. Inflammatory and Hematological Markers in Patients with Severe COVID-19

At 24 and 48 h after admission to the ICU, the D-dimer and ferritin concentrations were not significantly different, while LDH was significantly higher (*p* < 0.05) in non-survivors compared to survivors. At 24 h, IL-6 was significantly higher in non-survivors compared to survivors (*p* < 0.01). The median (Q1–Q3) IL-6 concentrations were 21.6 (9.7–55.4) and 35.1 (15.0–107.0) pg/mL for survivors and non-survivors, respectively. Table 2 presents the inflammatory markers in the overall population as well as in survivors and non-survivors from the ICU.

The hematological markers in the overall population as well as in survivors and non-survivors from ICU are shown in Table 3. At 24, 48, and 72 h after admission to the ICU, the MPV values were not significantly different between survivors and non-survivors. Significant decreases in lymphocyte counts were observed at 24, 48, and 72 h after ICU admission in non-survivors compared to survivors (*p* = 0.000), while the non-survivors showed significant increases in NLR values at 24, 48, and 72 compared to survivors (*p* = 0.000). The MPV/platelet ratios were higher in non-survivors compared to survivors at 48 and 72 h after ICU admission (*p* < 0.001).

### 3.3. Predictors of 28-Day Mortality

I the multivariate analysis, IL-6 values at 24 h ≥ 11, NLR values at 24 h ≥ 22, and NLR values at 72 h ≥ 14,were associated with 28-day mortality in patients with severe COVID-19 living at high altitude (*p* < 0.05). Consequently, we found that patients with severe COVID-19 with IL-6 values at 24 h ≥ 11, NLR values at 24 h ≥ 22, and NLR values at 72 h ≥ 14 were 8.3, 3.8, and 3.8 times more likely to die at 28 days, respectively (Table 4).

## 4. Discussion

The main finding of our study is that in mechanically ventilated patients admitted to the ICU with severe COVID-19 in low-resource settings and living at high altitude, low-cost and immediately available blood biomarkers such as IL-6 and NLR can predict mortality in the ICU.

A homogeneous population of severe mechanically ventilated COVID-19 patients admitted to the ICU was included in the analysis. Several inflammatory and hematological biomarkers were systematically analyzed at different timings from the ICU admission as potential predictors of the outcome. COVID-19 is a heterogeneous disease with high potential for multiple organ failure and impaired outcomes in patients admitted to an ICU. It has been hypothesized that the multisystem involvement in COVID-19 can be caused by an unbalanced immune response that facilitates the progression of the disease to multiple organs. This hypothesis has been confirmed by the presence of altered biomarkers and cytokines as the manifestation of inflammatory and thromboembolic disorders at high risk of progression to multiorgan failure [3]. Some biomarkers showed an optimal ability to predict the outcome in critically ill patients without COVID-19, but little is known about the specific biomarkers that can be used to predict survival in severe COVID-19. To the best of our knowledge, this is one of the first studies confirming the existence of low-cost and easily available prognostic biomarkers as predictors of mortality in this patient population.

In the present study, the patients with severe COVID-19 who died were older, more clinically severe, and with higher risk of organ failure (as measured by the APACHE-II and SOFA scores) than those who survived. Although there was a predominance of males over females as non-survivors as compared to survivors, the mortality rates did not differ by gender. This is in agreement with previous studies where age and severity were associated with worse clinical outcomes, with a predominance of male gender in the hospital admissions [19,20].

We found that pro-inflammatory markers such as IL-6 (at 24 h) and hematological markers such as NLR (at 24 and 72 h, respectively) were independent predictors of 28-day mortality in patients with severe COVID-19 living at high altitude in a low-resource setting. IL-6 is a pro-inflammatory cytokine that is produced by stromal cells and released by the activation of pro-inflammatory cytokines, especially IL-1β and tumor necrosis factor-α (TNF-α), and by lung macrophages after stimulation of toll-like receptors (TLR) [21]. In patients with non-COVID-19 ARDS, the persistent elevation of IL-6 levels was a consistent and efficient predictor of the outcome over time [22]. In COVID-19, an increase in IL-6 levels has been associated with the development of lung injury and hypoxemia, representing an important prognostic biomarker of severity [7,8,23]. However, the value of IL-6 as a predictor of the outcome in critically ill patients with COVID-19 is still controversial. Therefore, IL-6 has been proposed as a possible biological target for clinical and therapeutic decision-making, such as the use of IL-6 receptor blocker drugs [24]. Our results are in line with the available literature on the prognostic utility of this cytokine in patients with severe COVID-19 [7,8,23].

The severe inflammation observed in severe COVID-19 stimulates the production of neutrophils and induces apoptosis of the lymphocytes. This phenomenon is commonly observed in coronaviruses and Middle East respiratory syndrome coronavirus disease (MERS-CoV) infections, and the hematological change in leukocyte populations adequately predicted mortality [25]. In hospitalized patients with COVID-19, high neutrophil and low lymphocyte counts were independent predictors of mortality [26,27]. Some studies agree that by merging these two parameters into a unique biomarker (NLR), a more robust predictor of severity [14,28,29,30,31] and mortality at the time of ICU admission can be obtained [32]. The NLR has been recently investigated as a potential prognostic biomarker in COVID-19, influenza, and respiratory syncytial virus infections, showing significant associations with poor clinical outcomes only in patients with COVID-19 [33]. This suggests that the prognostic value of the NLR is specific to certain sub-populations of critically ill patients such as in COVID-19. A study from South America showed the better prognostic performance of NLRs > 5.5 over other inflammatory markers such as CRP, LDH, ferritin, and lymphocyte counts [34]. Furthermore, a retrospective study that analyzed more than 4000 patients living at high altitudes demonstrated a significant association of this marker with the severity and need for ICU admission [19]. Our results are consistent with the previous evidence [8,14,30,31,32,33,34], confirming the biological value of this biomarker, similarly to IL-6, which is a strong predictor of severity and mortality in COVID-19. Corticosteroids represent now a standard of care for critically ill patients with COVID-19 [35]. The effects include changes in blood cell counts, such as neutrophilia and lymphopenia, possibly explaining the increase in the NLR. In our series, the use of corticosteroids did not differ between non-survivors and survivors. Therefore, NLR changes can be attributed to the severity of COVID-19 and not to other confounding variables.

Platelets participate in the endothelial and thrombotic alterations of SARS-CoV-2 [36,37]. When dysregulated, platelets interact with neutrophils, forming neutrophil extracellular traps (NETs) to trigger immune–thrombosis and microcirculation disturbances. In COVID-19, the platelets are activated and aggregate chaotically, being consumed with possible increases in mean volume and decreases in the absolute count [26,38,39]. In our study, unlike other reports [10], the MPV was not associated with mortality. However, the MPV/platelet ratio was associated with mortality at 48 and 72 h, despite not being an independent predictor in the multivariate analysis. High altitudes seem to decrease the severity and mortality of SARS-CoV-2 infection [18,40]; however, the biological responses of biomarkers, especially those derived from blood counts, are different compared to at sea level. A preliminary study performed in a high-altitude city could not demonstrate the predictive performance of platelet counts, although they did not assess other platelet indices, in determining associations with mortality in critical patients with COVID-19 [8]. High altitudes are associated with a prothrombotic subphenotype [14]. At high altitudes, hypoxia generates hyperreactivity and increased platelet aggregation in response to adenosine diphosphate (ADP) and tends to increase other platelet indices [14]. This could explain the low predictive capacity of the MPV and the MPV/platelet ratio, which would allow us to hypothesize a lower performance rate for the platelet indices compared to that observed at sea level. However, the NLR, which is also derived from the blood count, has high prognostic potential for the prediction of mortality in critical patients with COVID-19. This is supposedly because unlike the platelets, lymphocytes, and neutrophils, the NLR does not participate in the coagulation but reflects inflammation only. In COVID-19, some validated scoring systems, such as the SCOPE score, which is based on biomarkers such as C-reactive protein (CRP), ferritin, D-dimer, and IL-6, demonstrated good prediction of the progression of COVID-19 pneumonia to severe respiratory failure or death within 14 days. This allowed therapeutic choices to be made, such as the administration of anakinra when the score was ≥6 [41]. In the present study, neither ferritin and D-dimer values nor scores of severity (APACHE-II and SOFA) could predict mortality in patients with severe COVID-19 living at high altitudes, so low-cost alternative markers such as NLR and IL-6 values could contribute to obtaining a score to predict mortality in this group of patients under these geographic conditions. The APACHE-II and SOFA scores are usually adopted to assess the severity of illness in critically ill patients. In our study, within this specific sub-population of COVID-19 patients, we sought to determine whether these scores have the same value for the prediction of the outcome as in critically ill subjects without COVID-19. We found that patients who had lower APACHE-II and SOFA scores died at similar rates to those with higher scores. Investigating this association in the multivariate analysis, we found that these scores seem to not be very effective for predicting mortality in critically ill patients with COVID-19. Therefore, we can assume that the APACHE-II and SOFA scores have differing prognostic value, depending on the population under study. Accordingly, some important features of COVID-19 that may be responsible for patients’ critical illnesses are not investigated by the APACHE-II and SOFA scores, including thromboembolic disorders.

Finally, the strengths of this research lie in the fact that it was determined that the IL-6 and NLR values, which are immediately available, low-cost, affordable laboratory markers in basic instrumentation laboratories, represent prognostic alternatives for critically ill patients with severe COVID-19 at high altitude in low-resource settings.

### Limitations

The present study has several limitations that need to be addressed. First, the retrospective design of the study limited the causative association with the outcome, and the study also lacked a validation sample. Second, only a few specific inflammatory and hematological markers are usually measured in high-, middle-, and low-income countries. We cannot exclude that other markers may be differently associated with the outcome. Third, the study population included patients with specific clinical characteristics admitted to the ICU and undergoing invasive mechanical ventilation, which would limit the extension of the present findings to different groups of COVID-19 patients with different disease severity levels. Fourth, we did not investigate COVID-19 variants that may have affected our findings, although our cohort was studied in a relative short period of time corresponding to the first peaks of the pandemic, when the beta and alpha variants were preponderant. Fifth, our findings are limited to a specific cohort of patients living at high altitude. Therefore, similar conclusions may not be obtained at sea level.

## 5. Conclusions

In the present retrospective study, low-cost markers such as IL-6 and the NLR could be used as potential predictors of the outcome in mechanically ventilated patients with severe COVID-19 admitted to the ICU living at high altitude. Further investigations are warranted to corroborate these findings.

## Figures and Tables

**Table 1:** Demographic and clinical characteristics in the overall population as well as in survivors and non-survivors in the intensive care unit. Data are expressed as medians (1st quartile (Q1)–3rd quartile (Q3)) or numbers (percentages). * Significant differences between survivors and non-survivors based on Student’s *t*-test ^1^, Chi-square test, or Fisher’s exact statistic ^2^ and Mann–Whitney U test ^3^. DM: diabetes mellitus; SOFA: sequential organ failure assessment; APACHE II: acute physiology and chronic health II.

Clinical Features	All Patients	Survivors	Non-Survivors	*p*-Value
	(*n* = 223)	(*n* = 145)	(*n* = 78)	
Median age (Q1–Q3), years ^1^	51 (26–75)	48 (25–72)	56 (31–79)	0.000 *
Sex, *n* (%) ^2^				
Male	157 (70.4)	99 (68.3)	58 (74.4)	0.343
Female	66 (29.6)	46 (31.7)	20 (25.6)	
DM, *n* (%) ^2^	28 (12.6)	16 (11.0)	12 (15.4)	0.350
Hypertension, *n* (%) ^2^	32 (14.4)	16 (11.0)	16 (20.5)	0.054
Obesity, *n* (%) ^2^	74 (33.2)	50 (34.5)	24 (30.8)	0.574
APACHE II, 24 h ^3^	16 (12–20)	16 (12–19.5)	18 (14–22)	0.010 *
SOFA ^3^				
24 h	7 (5–9)	7 (5–8)	8 (6–11)	0.001 *
48 h	5 (3–7)	5 (3–7)	7 (5–8)	0.000 *
72 h	4 (3–7)	4 (2–6)	6 (4–8)	0.000 *
Corticosteroid use, *n* (%) ^2^	201 (90.1)	131 (90.3)	70 (89.7)	0.886
Heparin use, *n* (%) ^2^	172 (77.5)	109 (75.2)	63 (81.8)	0.259
Hospitalization, days ^3^	10 (6–15)	10 (6–14)	12.5 (6.8–16.3)	0.129

**Table 2:** Inflammatory markers in the overall population, as well as in survivors and non-survivors in the intensive care unit. Data are expressed as medians (1st quartile (Q1)–3rd quartile (Q3)). * Significant differences between survivors and non-survivors based on Mann–Whitney U test; LDH: lactate dehydrogenase; IL-6: interleukin-6.

Inflammatory Markers	All Patients	Survivors	Non-Survivors	*p*-Value
	(*n* = 223)	(*n* = 145)	(*n* = 78)	
D-dimer 24 h, ng/mL	1161 (751.6–2684.5)	1055 (733.8–1910.8)	1318 (821.5–3257)	0.085
D-dimer 48 h, ng/mL	1227 (718–2704)	1221.5 (691.8–2099.2)	1311 (813–4290)	0.108
Ferritin 24 h, ng/mL	1137 (668.5–1650)	1040.5 (614.5–1650)	1348.5 (874.6–1650)	0.088
Ferritin 48 h, ng/mL	1140 (802–1500)	1075.8 (690.4–1500)	1187.1 (916.8–1500)	0.136
LDH 24 h, U/L	820 (671.5–1001.5)	773 (633–948)	887 (745.3–1103.3)	0.001 *
LDH 48 h, U/L	686.5 (579–859.5)	661 (559.8–820.8)	770 (624.5–910.5)	0.010 *
IL-6 24 h, pg/mL	25.2 (12.2–65.1)	21.6 (9.7–55.4)	35.1 (15.0–107.0)	0.001 *

**Table 3:** Hematological markers in the overall population, as well as survivors and non-survivors in the intensive care unit. Data are expressed as medians (1st quartile (Q1)–3rd quartile (Q3)). * Significant differences between survivors and non-survivors based on Mann–Whitney U test. MPV: mean platelet volume; NLR: neutrophil/lymphocyte ratio.

Hematology Markers	All Patients	Survivors	Non-Survivors	*p*-Value
	(*n* = 223)	(*n* = 145)	(*n* = 78)	
MPV, 24 h	8.9 (8.5–9.6)	8.9 (8.5–9.5)	8.9 (8.4–9.6)	0.650
MPV, 48 h	8.9 (8.5–9.4)	8.9 (8.5–9.4)	9 (8.5–9.6)	0.419
MPV, 72 h	9 (8.5–9.6)	8.9 (8.6–9.5)	9 (8.5–9.7)	0.502
Lymphocytes, 24 h (cells/mL)	620 (410–900)	660 (465–930)	465 (340–712.5)	0.000 *
Lymphocytes, 48 h (cells/mL)	520 (400–820)	620 (455–840)	455 (290–607.5)	0.000 *
Lymphocytes, 72 h (cells/mL)	555 (350–882)	630 (395–970)	430 (300–600)	0.000 *
NLR, 24 h	15.6 (9.6–23.4)	13.7 (8.4–20.1)	21.7 (12.7–33.1)	0.000 *
NLR, 48 h	15.6 (9.8–22.7)	13.1 (8.7–18.4)	22.0 (14.3–29.5)	0.000 *
NLR, 72 h	15.4 (9.1–25.9)	13.2 (7.8–21.3)	20.6 (14.1–31.7)	0.000 *
MPV/platelet, 24 h	2.8 (2.2–3.6)	2.7 (2.1–3.5)	2.9 (2.4–4.1)	0.052
MPV/platelet, 48 h	2.7 (2.1–3.6)	2.6 (2–3.3)	3.0 (2.3–4.3)	0.004 *
MPV/platelet, 72 h	2.7 (2.1–3.5)	2.5 (2.0–3.3)	3.1 (2.3–3.9)	0.003 *

**Table 4:** Multivariate regression model to predict mortality at 28 days in patients with severe COVID-19 admitted to the intensive care unit. * Mortality predictor variable, *p* < 0.05; ** significant risk CI does not include the value 1. MPV = mean platelet volume; NLR = neutrophil/lymphocyte ratio; CI = confidence interval; OR = odds ratio.

Variables	OR	95% CI	*p*-Value
SOFA 24 h ≥ 8	1.0	0.4–2.8	0.990
SOFA 48 h ≥ 6	1.1	0.3–3.8	0.825
SOFA 72 h ≥ 4	1.7	0.5–5.7	0.395
IL-6 24 h ≥ 11 **	8.3	1.5–44.6	0.014 *
LDH 24 h ≥ 781	1.7	0.6–4.4	0.301
LDH 48 h ≥ 709	2.0	0.7–5.6	0.180
NLR 24 h ≥ 22 **	3.8	1.3–10.9	0.015 *
NLR 48 h ≥ 18	0.8	0.3–2.5	0.746
NLR 72 h ≥ 14 **	3.8	1.3–11.0	0.013 *
MPV/Platelets 48 h ≥ 4	1.6	0.4–6.1	0.470
MPV/Platelets 72 h ≥ 3	1.4	0.5–4.0	0.480

## Data Availability

The data are available from the corresponding author under reasonable request.
